# Phosphorylated Tau protein in the myenteric plexus of the ileum and colon of normothermic rats and during synthetic torpor

**DOI:** 10.1007/s00441-020-03328-0

**Published:** 2021-01-29

**Authors:** R Chiocchetti, T Hitrec, F Giancola, J Sadeghinezhad, F Squarcio, G Galiazzo, E Piscitiello, M De Silva, M Cerri, R Amici, M Luppi

**Affiliations:** 1 Department of Veterinary Medical Sciences, Alma Mater Studiorum-University of Bologna, Bologna, Italy; 2 Department of Biomedical and Neuromotor Sciences, Alma Mater Studiorum-University of Bologna, Bologna, Italy; 3 Department of Medical and Surgical Sciences, Alma Mater Studiorum-University of Bologna, and St. Orsola-Malpighi Hospital, Bologna, Italy; 4 Department of Basic Sciences, Faculty of Veterinary Medicine, University of Tehran, Tehran, Iran

**Keywords:** Choline acetyltransferase, Enteric nervous system, Immunohistochemistry, Neuronal nitric oxide synthase, Tauopathies, Hypothermia

## Abstract

Tau protein is of primary importance for neuronal homeostasis and when hyperphosphorylated (PP-Tau), it tends to aggregate in neurofibrillary tangles, as is the case with tauopathies, a class of neurodegenerative disorders. Reversible PP-Tau accumulation occurs in the brain of hibernating rodents and it was recently observed in rats (a non-hibernator) during synthetic torpor (ST), a pharmacological-induced torpor-like condition. To date, the expression of PP-Tau in the rat enteric nervous system (ENS) is still unknown. The present study immunohistochemically investigates the PP-Tau expression in the myenteric plexus of the ileum and colon of normothermic rats (CTRL) and during ST, focusing on the two major subclasses of enteric neurons, i.e., cholinergic and nitrergic.

Results showed that both groups of rats expressed PP-Tau, with a significantly increased percentage of PP-Tau immunoreactive (IR) neurons in ST vs. CTRL. In all rats, the majority of PP-Tau-IR neurons were cholinergic. In ST rats, the percentage of PP-Tau-IR neurons expressing a nitrergic phenotype increased, although with no significant differences between groups. In addition, the ileum of ST rats showed a significant decrease in the percentage of nitrergic neurons. In conclusion, our findings suggest an adaptive response of ENS to very low core body temperatures, with changes involving PP-tau expression in enteric neurons, especially the ileal nitrergic subpopulation. In addition, the high presence of PP-Tau in cholinergic neurons, specifically, is very interesting and deserves further investigation. Altogether, these data strengthen the hypothesis of a common cellular mechanism triggered by ST, natural hibernation and tauopathies occurring in ENS neurons.

**Electronic supplementary material:**

The online version of this article (10.1007/s00441-020-03328-0) contains supplementary material, which is available to authorized users.

## Introduction

Tau protein (Tau) is a microtubule-associated protein that is predominantly present in neurons, where it is of primary importance for many physiological processes due to its effects on the dynamics of the microtubule system (Wang and Mandelkow 2016). When hyperphosphorylated (PP-Tau), Tau monomers detach from microtubules and tend to irreversibly aggregate in oligomers at first and in neurofibrillary tangles at a later time, as it occurs in a group of neurodegenerative disorders named tauopathies, such as Alzheimer’s disease (AD) or Parkinson disease (PD) (Gerson et al. 2016; Kovacs 2017).

However, accumulation of PP-Tau also occurs in the brain of hibernating rodents during hibernation bouts (Arendt et al. 2003, 2015; Stieler et al. 2011), as well as in mice during cold water stress (Okawa et al 2003), general anesthesia (Planel et al. 2007), or metabolically challenging conditions (Yanagisawa et al. 1999; Planel et al. 2001), considering that mice are facultative hibernators (Hudson and Scott 1979). Interestingly, in all the abovementioned cases, the PP-Tau accumulation is reversible, completely disappearing following the return to normal conditions. The same phenomenon has been described in the central nervous system (CNS) of a non-hibernating mammal (i.e., the albino rat) undergoing a pharmacologically induced torpor-like and deep hypothermic condition (Luppi et al. 2019), referred to as “synthetic torpor” (ST; Cerri 2017).

In sharp contrast to what is observed in the healthy adult brain, it has been shown that fetal brain neurons express PP-Tau, suggesting that degenerating neurons, during tauopathies, may lose their regulatory control of phosphorylation, resulting in the reappearance of a fetal phosphorylation pattern (Anderton et al. 1995). In line with this evidence, Lionnet et al. (2018) demonstrated that cultured fetal neurons of the rat “second brain,” i.e., the enteric nervous system (ENS), express constitutive PP-Tau protein and that their levels of phosphorylation can be downregulated and upregulated.

While it is known that the brain of adult rats, kept under normal laboratory conditions, does not express PP-Tau (Hu et al. 2017; Luppi et al. 2019), no data are available concerning the expression of PP-Tau in the ENS of adult rats. Although it is known that Tau can be phosphorylated at multiple molecular sites (Wang and Mandelkow 2016; Lionnet et al. 2018), in the present study we focus on Tau phosphorylated in Ser202/Thr205 by using a specific antibody (AT8) able to recognize the phosphorylation in both sites (Lionnet et al. 2018; Luppi et al. 2019). It is worth noting that this phosphorylation pattern is widely used in studies on tauopathies (Hu et al. 2017), also on humans in the post-mortem stage of neurofibrillary pathology in AD (Braak et al. 2006).

With this immunohistochemical approach, we aim to (1) identify PP-Tau (AT8) protein immunoreactivity (AT8-IR) in the myenteric plexus (MP) neurons of the adult rat ileum and colon, (2) verify whether AT8-IR is overexpressed in the ENS of rats undergoing a ST condition and (3) study whether a specific neuronal population of the ENS expresses AT8 immunolabeling, under control as well as ST conditions, by performing co-localization experiments with markers for the two major subsets of enteric neurons, i.e., cholinergic and nitrergic neurons, identified by the anti-choline acetyltransferase (ChAT) and anti-neuronal nitric oxide synthase (nNOS) antibodies, respectively.

Since the “little brain” can be a mirror of the “big brain” in both physiological and pathological conditions, data obtained in the present study may increase the knowledge on the role of PP-Tau in the rat ENS, particularly during deep hypothermia induced by ST.

## Material and methods

### Animals

The experimental design included 12 male Sprague–Dawley rats (201–225 g; Charles River), which were acclimated to normal laboratory conditions. The procedure to induce ST has been previously described (Cerri e al. 2013). Briefly, six deeply anesthetized rats (diazepam, 5 mg/kg i.m.; Ketamine–HCl, 100 mg/kg i.p.) placed in a stereotaxic apparatus (David Kopf Instruments; Tujunga, CA, USA) were surgically implanted with a microinjection guide cannula, targeted to the raphe pallidus, a key pontine nucleus in the neural pathway for thermogenic control (Morrison and Nakamura 2019). The tip of the guide cannula was placed at the following stereotaxic coordinates from the lambda: on the midline, 3.0 mm posterior and 8.0 mm ventral to the dorsal surface of the cerebellum (Paxinos and Watson 2007). Each rat recovered from surgery for at least 1 week. Prior to the experimental session, rats were placed in a cage positioned within a thermoregulated and sound-attenuated chamber. To induce ST, we used a consolidated protocol (Cerri et al. 2013; Luppi et al. 2019; Tinganelli et al. 2019). Briefly, a microinjecting cannula was inserted into the implanted guide cannula. Then, 100 nl of 1 mM muscimol (a GABA agonist) was injected once an hour, six consecutive times. Following the last injection, brain temperature reached values of around 22 °C (Cerri et al. 2013). As a control (CTRL) group, six animals were injected with artificial cerebrospinal fluid (aCSF; EcoCyte Bioscience). Rats were sacrificed/euthanized by decapitation under general anesthesia one hour following the last injection of muscimol, when the nadir of hypothermia was achieved (Cerri et al. 2013).

Fresh segments of ileum and colon were quickly removed from each animal and placed in phosphate-buffered saline (PBS: 0.15 M NaCl in 0.01 M sodium phosphate buffer, pH 7.2) containing the L-type calcium channel blocker nicardipine (10–6 M; Santa Cruz, SC-202731) to inhibit tissue contraction. The dissected pieces were opened along the mesenteric border and cleaned of their contents by being washed with PBS. They were then pinned out tautly, mucosa side down, onto a balsa-wood board and fixed overnight at 4 °C in 2% formaldehyde plus 0.2% picric acid in 0.1 M sodium phosphate buffer (pH 7.0). The next day, tissue was cleared of fixative with 3 × 10-min washes in 100% dimethylsulfoxide followed by 3 × 10-min washes in PBS. All tissue was stored at 4 °C in PBS containing sodium azide (0.1%).

### Immunohistochemistry

The mucosa, submucosa and circular muscle layers were removed to give longitudinal muscle-MP whole-mount. Tissue samples were preincubated at 4 °C in 2% Triton in PBS for 30 min (on an orbital shaker). After this preincubation, which appeared essential for obtaining a satisfactory AT8 immunostaining, tissues were preincubated in 10% normal donkey serum (NDS) in PBS containing 1% Triton X-100 and 1% bovine serum albumin (BSA) for 60 min at room temperature (RT) to reduce non-specific binding and to further permeabilize the tissue.

All primary antibodies (Table 1) were diluted in antibody diluent (1.8% NaCl in 0.01 M sodium phosphate buffer containing 0.1% sodium azide) and 10% NDS, 1% Triton X-100 and 1% BSA. Combinations of primary antibodies were used for double labeling. Following incubation in primary antibodies for two nights at 4 °C, preparations were given 3 × 10-min washes in PBS and then incubated for 3 h at RT with appropriate secondary antibodies (Table 2) diluted in 10% NDS in PBS containing 1% Triton X-100 and 1% BSA. Tissue was then further washed in PBS for 3 × 10- min and mounted with the DAPI Fluoroshield (F6057-20ML, Sigma Aldrich, Milan, Italy, Europe).

**Table 1:** Primary antibodies used in the study

Primary antibody	Host	Code	Dilution	Source
ChAT	Goat	AB144P	1:50	Millipore Sigma
HuC/HuD	Rabbit	ab210554	1:40	Abcam
HuC/HuD	Mouse	A-21271	1:200	Thermo Fisher
nNOS	Rabbit	GTX133407	1:100	Genetex
PGP9.5	Rabbit	NB300-675	1:200	Novus Biol
pTau (AT8)	Mouse	MN1020	1:200	Thermo Fisher

Primary antibody suppliers: Abcam, Cambridge, UK; Genetex, Irvine, CA, USA; Millipore Sigma, Burlington, MA, USA; Novus Biologicals, Littleton, CO, USA; Thermo Fisher Scientific, Waltham, MA USA

**Table 2:** Secondary antibodies used in the study

Secondary antibody	Host	Code	Dilution	Source
Anti-mouse IgG Alexa-488	Donkey	A-21202	1:250	Thermo Fisher
Anti-mouse IgG Alexa-594	Donkey	A-21203	1:250	Thermo Fisher
Anti-goat 594	Donkey	ab150132	1:400	Abcam
Anti-rabbit 594	Donkey	ab150076	1:1000	Abcam
Anti-rabbit 488	Donkey	A-21206	1:500	Thermo Fisher

Secondary antibody suppliers: abcam, Cambridge, UK; Thermo Fisher Scientific, Waltham, MA USA

Neurons were identified with the mouse anti-HuC/HuD and rabbit anti-HuC/HuD antibodies and with the rabbit anti-PGP9.5 antibody; general nucleus counterstaining was obtained by DAPI incubation.

Since the mouse and rabbit anti-HuC/HuD antibodies were raised against different antigens (HuC/HuD from human and zebrafish proteins, respectively), we tested these two antibodies on tissues of CTRL and ST rats. Co-localization experiments showed that the two anti-HuC/HuD antibodies identified the same neuronal cell bodies in tissues of CTRL rats, whereas in tissues of ST rats only the mouse anti-HuC/HuD antibody was able to adequately identify all the neurons. In tissues of hypothermic rats, the neuronal immunolabeling of the rabbit anti-HuC/HuD antibody was weak and not always identifiable. The rabbit anti-PGP9.5 antibody also failed to recognize all the enteric neurons in the MP whole-mount preparations of hypothermic rats. In MP ganglia, DAPI-labeled neuronal nuclei were easily recognizable from the nuclei of enteric glial cells and smooth muscle cells, characterized by their smaller dimension and stronger brightness and by the typically elongated and narrow nuclei, respectively. Since HuC/HuD/DAPI and PGP9.5/DAPI co-localization preliminary experiments demonstrated that 95% of the HuC/HuD and 97% of the PGP9.5 immunolabeled MP neurons (200 neurons counted for each marker) were also clearly identifiable with DAPI alone (Supplementary Fig. 1), DAPI was also used as supplementary/alternative neuronal staining.

**Fig. 1:**
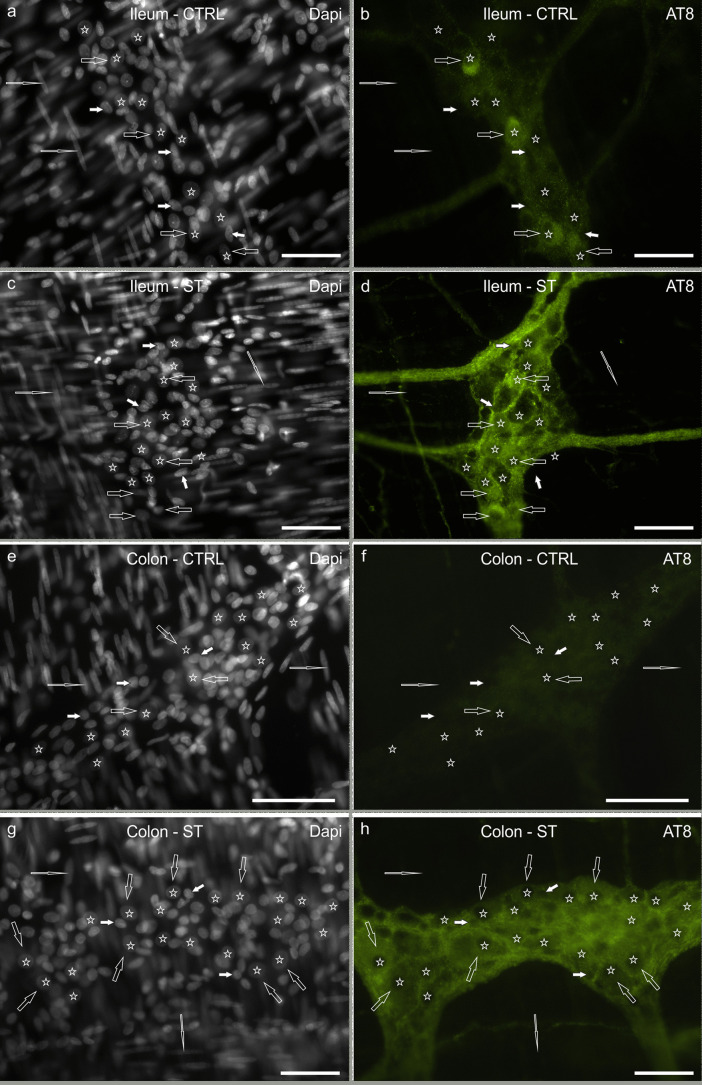
**a**–**h** Photomicrographs of whole-mount preparations of myenteric plexus (MP) of rat ileum (**a**–**d**) and colon (**e**–**h**) showing AT8 immunolabeling in control normothermic (CTRL) rats (**a**, **b**; **e**, **f**) and hypothermic (synthetic torpor, ST) rats (**c**, **d**; **g**, **h**). Open arrows indicate MP neurons that showed moderate-to-bright AT8 immunoreactivity in the ileum of CTRL rats (**b**) and faint AT8 immunoreactivity in the colon (**f**). In the ST rats, MP neurons (open arrows) showed bright AT8 immunoreactivity in both the intestinal segments (**d**, **h**). Stars indicate some of the neuronal nuclei stained with DAPI (**a**, **c**, **e**, **g**), which were recognizable for their shape, dimension and staining intensity. White arrows indicate the nuclei of some glial cells, which were smaller than the neuronal nuclei. Elongated open arrows indicate the nuclei of some smooth muscle cells. Bar: **a**–**h** = 50 µm

For this reason, the proportions of AT8-IR neurons were calculated by using DAPI labeling of the neuronal nuclei in both groups (CTRL and ST) of rats, while the proportions of ChAT- and nNOS-IR neurons were calculated by using the pan-neuronal marker mouse anti-HuC/HuD antibody.

### Fluorescence microscopy

Preparations were examined on a Nikon Eclipse Ni microscope equipped with the appropriate filter cubes to distinguish the fluorochromes employed. The images were recorded with a Nikon DS-Qi1Nc digital camera and NIS Elements software BR 4.20.01 (Nikon Instruments Europe BV, Amsterdam, Netherlands). Enteric neuron counts were performed at ×40 magnification. Slight adjustments to contrast and brightness were made using Corel Photo Paint, whereas the figure panels were prepared using Corel Draw (Corel Photo Paint and Corel Draw, Ottawa, ON, Canada).

### Quantitative analysis

The proportions of neurons that were HuC/HuD-IR or DAPI labeled and that were also immunoreactive for AT8, nNOS, or ChAT were determined by examining fluorescently labeled, double-stained preparations. Neurons were firstly localized/identified either by the presence of a fluorophore labeling one antigen (HuC/HuD) or by DAPI labeling and afterwards, the microscope filter was switched to determine whether or not the neuron expressed a second antigen (AT8, nNOS, or ChAT), identified with a different-colored fluorophore. In doing so, proportions of neurons labeled for pairs of antigens were determined.

To calculate the proportions of AT8-, ChAT- and nNOS-IR neurons, a minimum of 100 HuC/HuD-IR or DAPI-labeled neurons were counted from each animal. For co-localization studies, a minimum of 50 immunolabeled cells were counted for each animal. The percentages of immunolabeled neurons were calculated and expressed as mean ± standard deviation, with *n* being the number of animals considered.

Quantitative analysis of AT8-IR intensity was performed for both the ileum and colon of *n* = 4 CTRL and *n* = 4 ST rats. For each animal and enteric tract, three randomly selected ganglia were acquired (high magnification, ×40) using the same exposure times and were analyzed using ImageJ software (https://imagej.nih.gov/ij/). Standardized thresholds for brightness and contrast were determined empirically and applied to all images. Signal intensity was measured for each ganglion in arbitrary units and the obtained value was normalized for the ganglionic area considered.

### Statistical analysis

The data related to the density of AT8-, ChAT- and nNOS-IR neurons obtained in control-rats were compared with those obtained in ST rats. Due to the small number of subjects, we could not consider a Gaussian distribution for each set of data; as a consequence, the Mann–Whitney (MW) nonparametric test was used to analyze the differences between the two groups of rats (CTRL vs. ST). The level of significance was set at *P* < 0.05. The same criteria were applied to analyze differences in terms of AT8-IR intensity between the two groups. All analyses and graphical representations were performed using a commercial software (GraphPad Prism version 5.00 for Windows, GraphPad Software Inc., La Jolla, CA, USA).

## Results

### AT8-immunoreactive neurons in the ileum

CTRL rats: unexpectedly, a large number of nerve cell bodies showed cytoplasmic AT8-IR (41 ± 6%; 800/1951 cells counted, *n* = 4) (Fig. 1a, b). The mean number of total myenteric neurons per ganglion was 20 ± 2. The intensity of AT8 immunolabeling, which appeared homogeneously distributed throughout the cytoplasm, varied considerably (from weak to moderate) between neurons and only in some cases neurons showed bright granular AT8-IR. The medium- and large-sized AT8 immunoreactive neurons showed, in general, a smooth outline and were oval in shape, resembling sensory Dogiel type II neurons (Furness et al. 2006). Among small AT8-IR nerve cells, it was difficult to detect neurons showing an irregular outline (Dogiel type I neurons; Furness et al. 2006) but it cannot be excluded that some small AT8-IR neurons might have shown Dogiel type I morphology. Intra-ganglionic and inter-ganglionic nerve fibers and tertiary plexus nerve fibers (i.e., those fibers reaching the longitudinal muscle layer) showed moderate AT8-IR.

ST rats: in hypothermic rats, the percentage of MP neurons showing AT8-IR was 60 ± 6% (999/1664 cells counted, *n* = 4) (Fig. 1c, d). The mean number of total myenteric neurons per ganglion was 23 ± 5. The granular AT8 immunolabeling of the ganglionic neuropil was apparently more intense than in CTRL and it was sometimes so bright as to hamper the count of the positive neurons. Furthermore, the nerve fibers outside the ganglia showed bright AT8-IR (Fig. 1c, d).

**Fig. 2:**
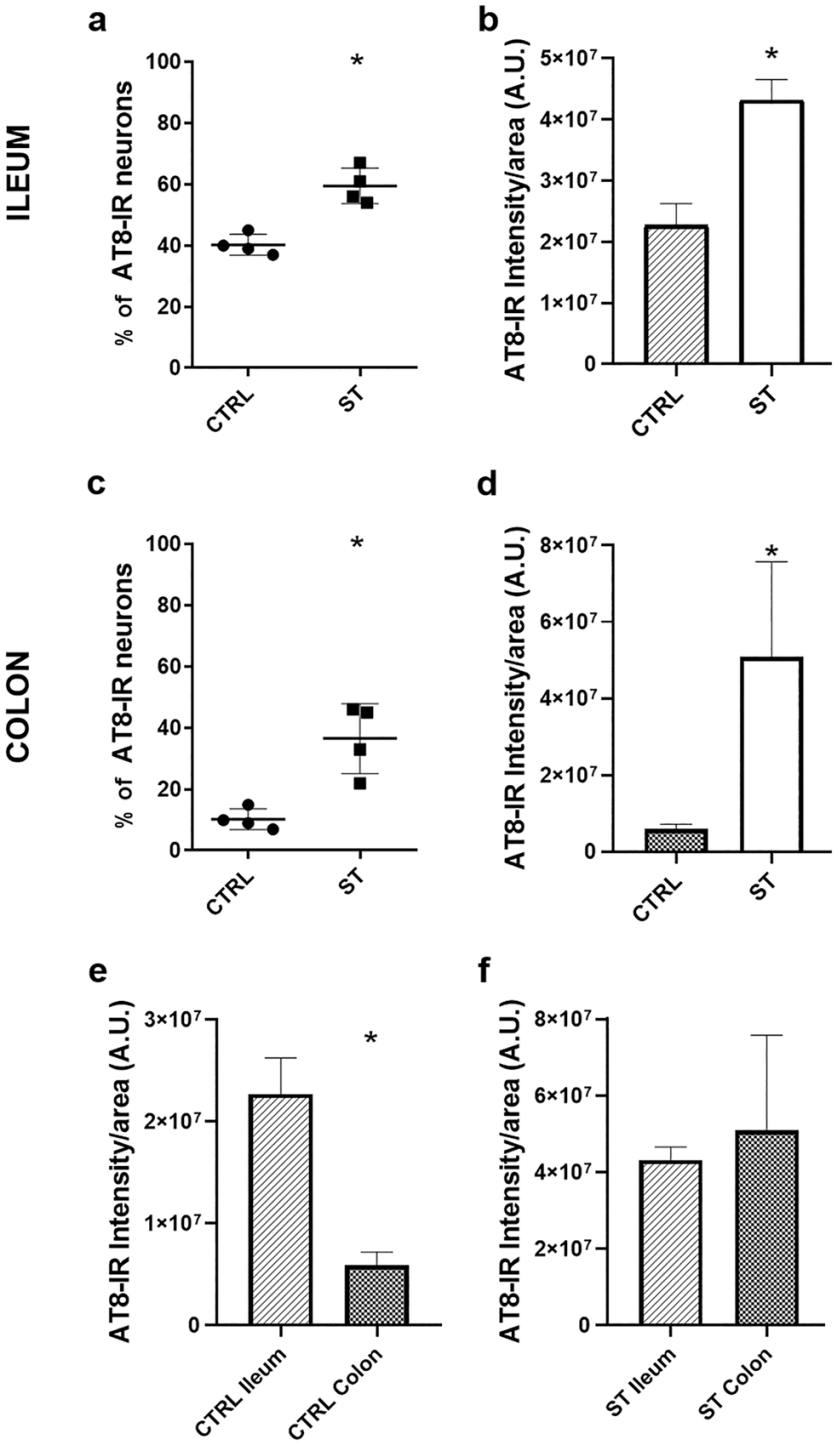
Graphical representation of the percentages of AT8 immunoreactive (-IR) neurons considering the total number of neurons counted in the ileum (**a**) and colon (**c**) of normothermic (CTRL) and hypothermic (synthetic torpor, ST) rats; AT8 signal intensity in arbitrary unit (A.U.) for ganglionic area in the ileum (**b**) and colon (**d**) of CTRL and ST rats; AT8 signal intensity for ganglionic area in ileum vs. colon in the two different experimental conditions (**e**) CTRL rats and (**f**) ST rats. Data are represented as mean ± SD (*P < 0.05)

Statistical analysis confirmed that, in the ileum of ST vs. CTRL rats, there was a significant increase in the AT8 signal both in terms of percentage of AT8-IR neurons (*P* = 0.03) and in terms of AT8-IR signal intensity per ganglionic area (*P* = 0.03) (Fig. 2a, b).

### AT8-immunoreactive neurons in the colon

CTRL rats: in the colon, the AT8-IR was very faint or nearly undetectable (Fig. 1e, f). The percentage of AT8-IR neurons was 10 ± 3% (176/1471 cells counted, *n* = 4). The mean number of total myenteric neurons per ganglion was 27 ± 3. Intra-ganglionic and inter-ganglionic nerve fibers showed very faint AT8-IR, whereas the tertiary plexus nerve fibers were not visible.

ST rats: in hypothermic rats, the MP neurons and nerve fibers showed bright AT8-IR (Fig. 1g, h). The percentage of AT8-IR neurons was 37 ± 11% (764/2087 cells counted, *n* = 4). The mean number of total myenteric neurons per ganglion was 30 ± 8. The pattern of AT8 immunolabeling in neurons and nerve fibers was similar to that observed in the ileum.

The statistical analysis confirmed that in the colon of ST vs. CTRL rats there was a significant increase in the AT8 signal, both in terms of percentage of AT8-IR neurons in ST vs. CTRL rats (*P* = 0.03) and in terms of AT8-IR signal intensity per ganglionic area (*P* = 0.03) (Fig. 2c, d).

Data emerging from quantitative analysis of AT8-IR intensity showed that, in CTRL rats, a significant difference existed between the two segments considered, with the ileum having a stronger AT8 signal compared with the colon (*P* = 0.03; Fig. 2e). In the ileum and colon of ST rats, no difference in AT8-IR intensity was detected (*P* > 0.99; Fig. 2f).

### ChAT- and nNOS-immunoreactive neurons in the ileum

The morphology and distribution of ChAT and nNOS immunoreactive neurons conformed to previous descriptions in the rat ileum (Mann et al. 1999). In short, the weak-to-moderate ChAT-IR was cytoplasmic and the great majority of ChAT immunoreactive neurons had a Dogiel type II morphology; their nerve processes were poorly stained. Nitrergic neurons, which mainly showed Dogiel type I morphology, expressed, overall, a brighter immunolabeling.

CTRL rats: in normothermic rats, the percentages of ChAT- and nNOS-IR neurons were 69 ± 6% (303/433 cells observed; *n* = 3) and 26 ± 4% (119/483 cells observed; *n* = 3), respectively (data not shown). The percentages of cholinergic and nitrergic neurons are consistent with those recently reported in other studies on the rat ileum (see “Discussion”).

ST rats: in hypothermic rats, there was a moderate-to-remarkable decrease in the fluorescence of ChAT and nNOS immunoreactivity. In addition, there were areas of whole-mount preparations in which nNOS immunolabeling was very faint or undetectable. Conversely, ChAT immunolabeling, although reduced, was recognizable in all the ganglia.

The percentages of ChAT- and nNOS-IR neurons were 69 ± 11% (553/778 cells observed; *n* = 6) and 20 ± 3% (364/1695 cells observed; *n = *5), respectively (data not shown).

**Fig. 3:**
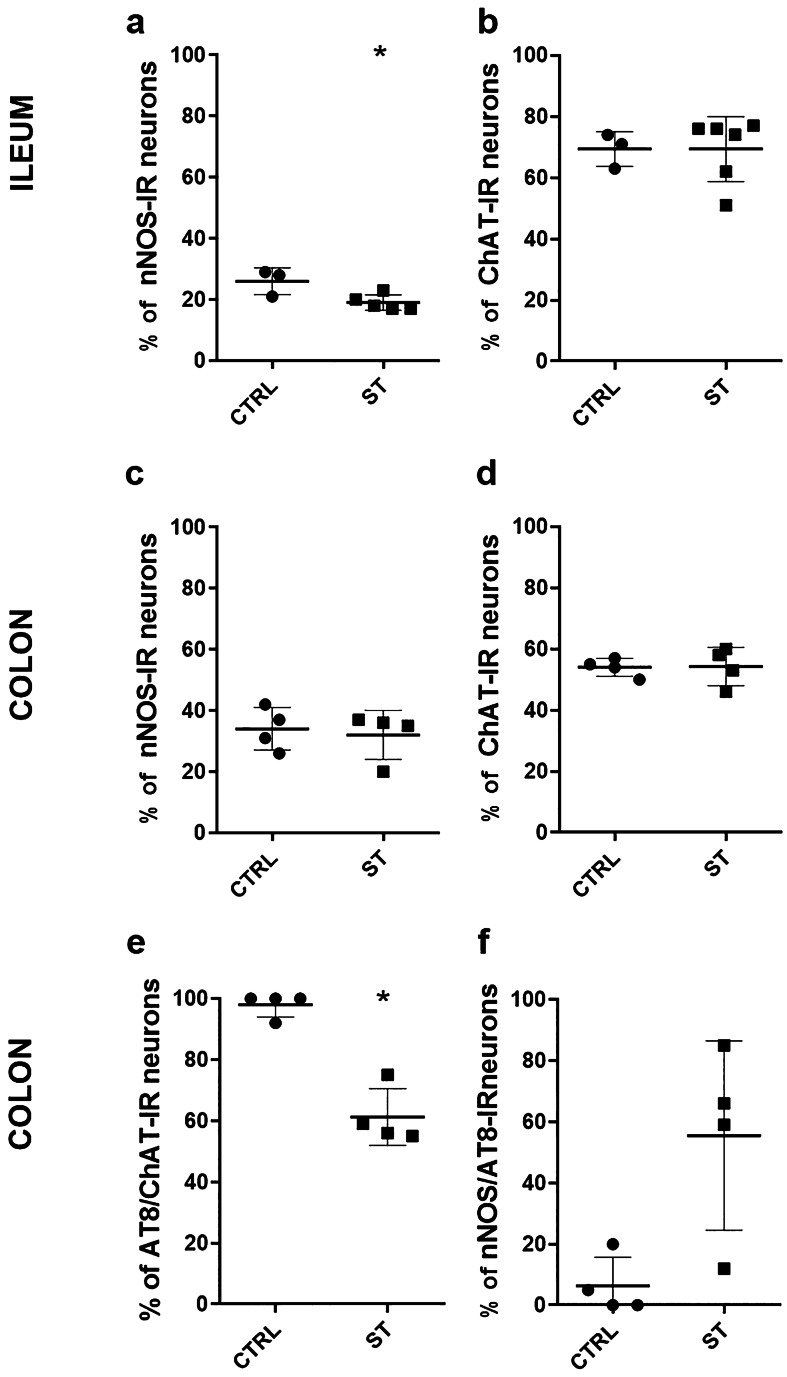
Scatter dot plots representing the percentages of nNOS-IR neurons and ChAT-IR neurons considering the total number of neurons counted in the ileum (**a**, **b**) and colon (**c**, **d**) of normothermic (CTRL) and hypothermic (synthetic torpor, ST) rats. Percentages of AT8 immunoreactive neurons co-expressing ChAT-IR (**e**) and percentages of nNOS-IR neurons co-expressing AT8 in the colon of CTRL and ST rats (**f**). Data are represented as mean ± SD (*P < 0.05)

Statistical analysis highlighted a significant decrease in the percentage of nNOS-IR neurons in the ileum of ST vs. CTRL rats (*P* = 0.05) (Fig. 3a), while no differences were observed in the percentage of ChAT-IR neurons between the two groups (*P* = 0.6; Fig. 3b).

### ChAT- and nNOS-immunoreactive neurons in the colon

CTRL rats: in the colon of normothermic rats, the percentages of ChAT- and nNOS-IR neurons were 54 ± 3% (205/376 cells observed; *n* = 4) and 34 ± 7% (329/965 cells observed; *n* = 4), respectively.

ST rats: unlike what had been observed in the ileum, in hypothermic rats an increase in the fluorescence of ChAT and nNOS immunoreactivity was seen; in addition, nNOS immunolabeling was detectable in every ganglion. The percentages of ChAT- and nNOS-IR neurons were 54 ± 6% (388/722 cells observed; *n* = 4) and 32 ± 8% (309/949 cells observed; *n* = 4), respectively (data not shown).

From statistical analysis no differences in the percentage of nNOS-IR neurons between ST vs. CTRL rats emerged (*P* = 0.7; Fig. 3c), nor were differences revealed in the percentages of ChAT-IR neurons between the two groups (*P* = 0.9; Fig. 3d).

## Co-localization studies

### AT8/ChAT immunoreactivity in the ileum

**Fig. 4:**
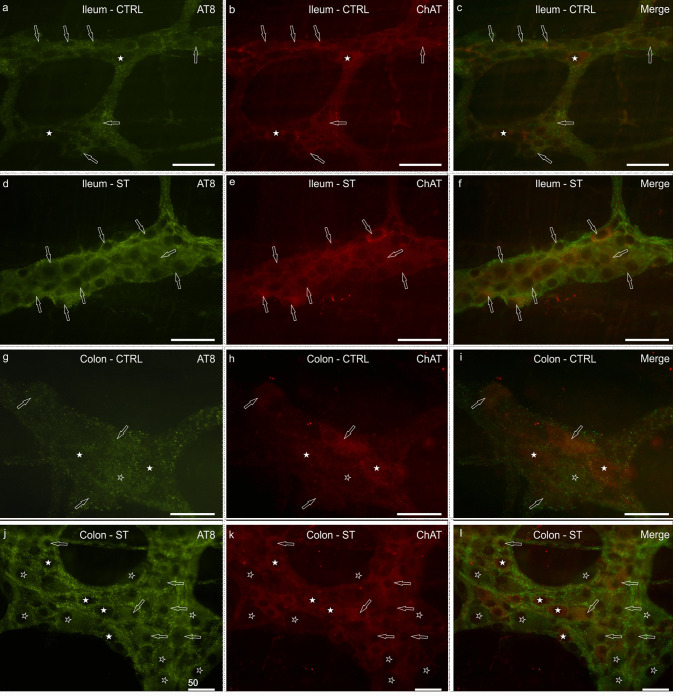
Photomicrographs of whole-mount preparations of myenteric plexus (MP) of the ileum (**a**–**f**) and the colon (**g**–**l**) of normothermic (CTRL) (**a**–**c**; **g**–**i**) and hypothermic (synthetic torpor, ST) rats (**d**–**f**; **j**–**l**) showing AT8 and choline acetyltransferase (ChAT) immunolabeling. Open arrows indicate MP neurons co-expressing AT8 (**a**, **d**, **g**, **j**) and ChAT (**b**, **e**, **h**, **k**) immunoreactivity. White stars indicate cholinergic neurons, which were AT8 negative. Open stars (**g**–**l**) indicate AT8 immunoreactive neurons, which were ChAT negative. In (**c**, **f**, **i**) and (**l**), the merge images. Bar: (**a**–**l**)= 50 µm

CTRL rats: in normothermic rats, the majority (95 ± 2%; 199/211 cells counted, *n* = 3) of AT8 immunoreactive neurons co-expressed ChAT-IR (Fig. 4a–c); among the cholinergic neurons, those co-expressing AT8-IR were 67 ± 4% (203/304 cells counted, *n* = 3).

ST rats: in hypothermic rats, the percentage of AT8 immunoreactive neurons co-expressing ChAT-IR was 87 ± 8%; 214/256 cells counted, *n* = 3) (Fig. 4d–f); among the cholinergic neurons, those which co-expressed AT8-IR in ST rats were 65 ± 8% (204/312 cells counted, *n* = 3).

Statistical analysis indicated that there was not a significant difference between the percentages of AT8-IR neurons co-expressing ChAT-IR in ST vs. CTRL rats (*P* = 0.20), neither was any difference identified between the percentages of ChAT-IR neurons co-expressing AT8-IR (*P* = 0.99) (data not shown).

### AT8/ChAT immunoreactivity in the colon

CTRL rats: since the identification of AT8-IR neurons in normothermic rats was very challenging, the minimum number of neurons co-expressing AT8- and ChAT-IR was not reached. However, the majority (98 ± 2%; 35/36 cells counted, *n* = 4) of AT8 immunoreactive neurons co-expressed ChAT-IR (Fig. 4g–i); among the cholinergic neurons, those co-expressing AT8-IR accounted for 18 ± 6% of the total (36/205 cells counted, *n* = 4).

ST rats: in hypothermic rats, the percentage of AT8 immunoreactive neurons co-expressing ChAT-IR was 61 ± 9%; 142/243 cells counted, *n* = 4) (Fig. 4j–l); among the cholinergic neurons, 42 ± 23% co-expressed AT8-IR in ST rats (195/457 cells counted, *n* = 4).

Statistical analysis indicated that there was a significant reduction in the percentages of AT8-IR neurons co-expressing ChAT-IR in ST vs. CTRL rats (*P* = 0.03; Fig. 3e), while no difference in the percentages of ChAT-IR neurons co-expressing AT8-IR was detected (*P* = 0.1) (data not shown).

### AT8/nNOS immunoreactivity in the ileum

**Fig. 5:**
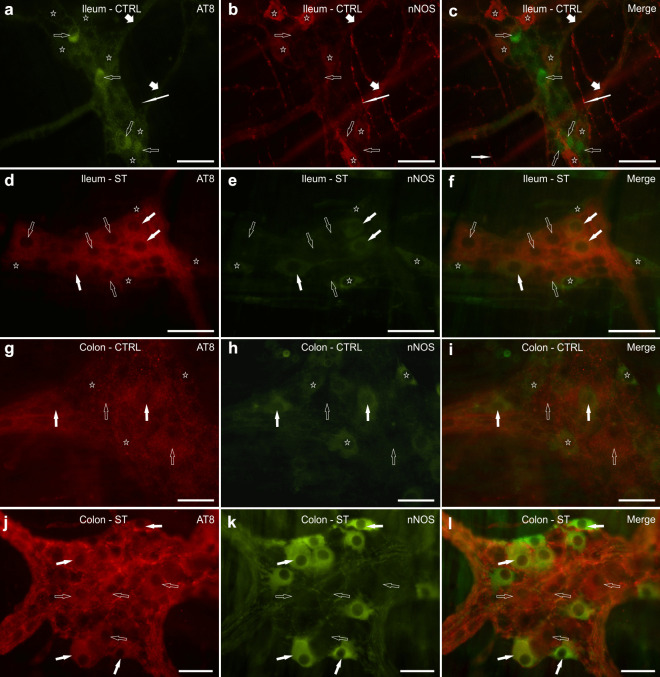
Photomicrographs of whole-mount preparations of myenteric plexus (MP) of the ileum (**a**–**f**) and the colon (**g**–**l**) of normothermic (CTRL) (**a**–**c**; **g**–**i**) and hypothermic (synthetic torpor, ST) rats (**d**–**f**; **j**–**l**) showing AT8 (**a**, **d**, **g**, **j**) and neuronal nitric oxide synthase (nNOS) (**b**, **e**, **h**, **k**) immunolabeling. Open arrows indicate MP neurons expressing AT8 (**a**, **d**, **g**, **j**) immunoreactivity. Stars indicate nitrergic neurons, which were AT8 negative. White arrows (**d**–**l**) indicate some MP neurons co-expressing AT8 and nNOS immunoreactivity. Long white arrows (**a**–**c**) indicate nitrergic nerve fibers, which were AT8 negative. In (**c**, **f**, **i)** and ( **l** ) are the merge images. Bar: (**a**–**l** ) = 50 µm

CTRL rats: only a small percentage of AT8 immunoreactive neurons co-expressed nNOS-IR (8 ± 2%, 25/309 cells counted, *n* = 3) (Fig. 5a–c); among the nitrergic neurons, those co-expressing AT8-IR were 12 ± 7% (27/259 cells counted, *n* = 3). The nNOS immunoreactive intra- and inter-ganglionic nerve fibers only showed a poor co-localization with AT8 immunolabeling, whereas nitrergic nerve fibers of the tertiary plexus were AT8 negative.

ST rats: as already seen for the calculation of the proportions of nitrergic neurons in ST rats (see above), in this case we also encountered difficulties in detecting nNOS immunolabeling.

In hypothermic rats, the percentage of AT8 immunoreactive neurons co-expressing nNOS-IR was 22 ± 6% (72/295 cells counted, *n* = 3) (Fig. 5d–f); among the nitrergic neurons, those co-expressing AT8-IR were 47 ± 12%, 87/187 cells counted, *n* = 3.

Statistical analysis indicated that in ST vs. CTRL rats, there was not a significant difference between the percentages of AT8-IR neurons co-expressing nNOS-IR (*P* = 0.10), neither was there between the percentages of nNOS-IR neurons co-expressing AT8-IR (*P* = 0.10) (data not shown).

### AT8/nNOS immunoreactivity in the colon

CTRL rats: the percentage of AT8 immunoreactive neurons co-expressing nNOS-IR ranged between 0 and 53% (average 19%) (23/96 cells counted, *n* = 4) (Fig. 5g–i); among the nitrergic neurons, those co-expressing AT8-IR were 6 ± 9% of the total (23/329 cells counted, *n* = 4).

ST rats: in hypothermic rats, the percentage of AT8 immunoreactive neurons co-expressing nNOS-IR was 50 ± 18% (148/315 cells counted, *n* = 4) (Fig. 5j–l); among the nitrergic neurons, those co-expressing AT8-IR were 56 ± 31% (178/329 cells counted, *n* = 4).

Statistical analysis indicated that in ST vs. CTRL rats, there was no difference between the percentages of AT8-IR neurons co-expressing nNOS-IR (*P* = 0.1), while the percentages of nNOS-IR neurons co-expressing AT8-IR were slightly increased in ST vs CTRL rats, though not significantly (*P* = 0.06; Fig. 3f).

## Discussion

Tau protein and mechanisms underlying its hyperphosphorylation represent an intriguing issue in the research field of neurodegeneration, especially due to the fact that this posttranslational modification is associated with human CNS disorders. As a consequence, accumulation of PP-Tau in CNS neurons of animal models have already been explored under specific conditions, such as hibernation, cold water stress, general anesthesia, or metabolically challenging conditions, while in the ENS, evidence of Tau hyperphosphorylation has still been poorly investigated.

In 2009, Phillips et al. identified a Tau protein hyperphosphorylated on the Ser262 in the dystrophic alpha-synuclein axons and terminals of the intestine of aged rats. At present, in human ENS neurons, only the non-phosphorylated Tau protein form has been observed (Deguchi et al. 1993; Tam and Owen 1993). The present study is the first showing the presence of hyperphosphorylated (Ser202/Thr205) Tau protein (Lionnet et al.^2018^; Luppi et al. 2019) in the enteric neurons of adult rats.

In the CNS, the hyperphosphorylation of Tau protein is, together with the amyloid beta (Aβ) peptide deposit, a hallmark of neurodegenerative diseases such as AD. Although the ENS may represent, in healthy and diseased conditions, a mirror of the CNS, studies of the human ENS in AD have been sparse (Joachim et al. 1989; Shankle et al. 1993). Animal models of AD have been used to investigate the progression of AD in the gut as well (Van Ginneken et al. 2011; Manocha et al. 2019).

The expression of a constitutive PP-Tau (AT8) in the intestinal intramural neurons of the rat acquires a certain theoretical importance since recent works have indicated a potential for cell-to-cell transmission of abnormally aggregated proteins such as Tau (Godert et al. 2014; Ahmed et al. 2014; Sanders et al. 2014; Clavaguera et al. 2015). In addition, in the human brain, the presence of PP-Tau in neuroanatomically connected brain regions has long suggested a physical spread of disease scheme.

Due to the technical precautions necessary to immunohistochemically identify a convincing/brilliant signal of AT8-IR in the rat ileum and colon (see above), it cannot be ruled out that the presence of PP-Tau in enteric neurons of other species, including humans, has been to date underestimated.

Enteric neuronal phenotypic diversity is extensive and virtually every class of neurotransmitters found in the CNS has also been detected in the ENS (Rao and Gherson 2016). Besides the presence of AT8 in ENS of adult rats, our findings indicated a great percentage of constitutive PP-Tau (AT8) in the MP cholinergic neurons of the ileum; even though this result is fascinating, at present we are not able to give a trustable explanation and we can only speculate that when this modification of Tau protein occurs, it is related to cholinergic neurons function. In line to what was described in the CNS (Luppi et al. 2019), in the colon of control rats we observed a moderate/low presence of the constitutive PP-Tau (AT8). This finding appears in contrast to what was found in the ileum, in which the constitutive presence of the protein is higher. However, it has to be noted that cholinergic neurons in the CNS represent the most affected neuronal subpopulation in human AD (Schliebs et al. 2005; Chen and Mobley 2019). In addition, even during hibernation, cholinergic neurons seem to be somehow functionally affected, as recently observed by Bullmann et al. (2019) in the hamster. Since acetylcholine plays many roles in central and peripheral cholinergic neurotransmission, in neuromuscular communication and also in interacting with other neurotransmitter pathways, cholinesterase inhibitors are a mainstay of AD therapy (Nordberg et al. 2009). However, the distinctive pathological alterations taking place in the “second brain” of AD patients are still largely undefined (Han et al. 2017).

In a murine model of AD (APP/PS1 mouse), in which the typical pathological features (deposition of Aβ and PP-Tau) of AD also occur in the ENS (Feng et al. 2018), a significant decrease of both ChAT and nNOS immunoreactive neurons has been observed (Han et al. 2017).

In the ileum of ST rats, the ChAT and nNOS immunolabeling was reduced in terms of brightness, when compared with controls, whereas in the colon of ST rats the signal of the two markers was enhanced. At present, this discrepancy is yet unclear; it is plausible that the ENS of the two different intestinal tracts, displaying distinct structures and functions, react in different ways to the hypothermic condition; a hypothesis that is also supported by the significant decrease in the proportion of nitrergic neurons in the ileum of ST rats.

We can reasonably exclude the hypothesis that the reduction of nitrergic neurons in ST is the consequence of oxidative stress, to which nitrergic neurons are particularly susceptible (Rivera et al. 2011): indeed, at temperatures that are far below the euthermic level, metabolism and oxygen consumption are dramatically reduced (Heldmaier et al. 2004). A possible explanation of the loss of nNOS immunoreactivity during ST is provided by Röszer and colleagues (2004), who reported that snails, during hypothermic resting periods, show a reduction in nNOS expression in enteric neurons.

Concerning the proportions of ChAT (69 ± 6%) and nNOS (26 ± 4%) immunoreactive neurons in the ileum of CTRL rats, our findings are consistent with those available in the literature. In fact, studies on rat ileum indicate that ChAT immunoreactive neurons account for about 75% of the entire MP neuronal population (Mann et al. 1999; Brasileiro et al. 2019), whereas nitrergic neurons account for 23 to 30% of the entire neuronal population (Nichols et al. 1993; Lin et al. 2011; Mann et al. 1999; Brasileiro et al. 2019). In the colon of CTRL rats, the percentage of nitrergic neurons (34 ± 7%) is consistent with the value (about 34%) recently reported by Da Silva et al. (2015), whereas the percentage of cholinergic neurons (54 ± 3%) was reduced in comparison with that (about 70%) reported by the same authors (Da Silva et al. 2015).

Another interesting result of this study was the significant increase in the AT8 signal in the enteric neurons of ST rats.

The neuronal PP-Tau expression is also a reversible hallmark observed in the CNS of hibernating animals (Arendt et al. 2015) as well as in rats under ST (Luppi et al. 2019). The accumulation of PP-Tau (AT8) in hypothermic conditions may depend on the characteristics of the main enzymes involved in the phosphoregulation of Tau protein: glycogen-synthase kinase-3-β (GSK3-β) and protein-phosphatase-2A (PP2A) (Planel et al. 2004;Gerson et al. 2016). It could be interesting, if not even necessary, to study the functional activity of these enzymes in the ENS of normothermic and ST rats, as well.

With regard to what was observed in the CNS (Luppi et al. 2019), the phosphorylation pattern observed in the ENS differs under normal conditions but is similar during ST. Since the phosphorylation of Tau protein is mandatory for neuronal plasticity (Wang and Mandelkow 2016), the constitutive ileal PP-Tau found in the present work may support the hypothesis by Kulkarni and colleagues (2017): in the ENS of the small intestine of adult mice, frequent neurogenesis occurs. Considering our results obtained from the colon, the process of neurogenesis might not be homogeneous throughout the enteric tract, as it is probably increased (as it possibly occurs in an augmented manner) in the ileum; however, to the best of our knowledge, no comparative studies on neurogenesis in the small and large intestine are yet available. However, ST induces a higher accumulation of PP-Tau in both ENS and CNS. As discussed in Luppi et al. (2019), since the pattern of PP-Tau accumulation observed in rats is similar to those shown in mammals that evolved the ability to hibernate, this process appears to be the outcome of a phylogenetically well conserved biochemical pathway, possibly aimed at protecting neurons from a very cold environment. From a functional point of view, whether these changes interfere with normal enteric function is not known and should be the focus of future experiments.

In addition, considering the reversibility of PP-Tau accumulation in the CNS neurons of hibernating mammals and in ST, it could be interesting to verify whether this process is reversible in the ENS as well, also in respect with the timing observed in the CNS.

Finally, a limitation of the study might be represented by the use, as CTRL rats, of animals which underwent surgical and pharmaceutical treatments before their suppression. However, a pilot study (data not published) showed AT8-IR also in the ENS neurons of the ileum of rats that never were subjected to surgery and in addition, brain sections of CTRL rats never presented AT8-IR (Hu et al. 2017; Luppi et al. 2019).

## Conclusion

In the present research, the expression of constitutive PP-Tau (AT8) immunolabeling has been described in the intestine of adult rats for the first time. In addition, the induction of a widespread AT8 immunolabeling has been also described in the ENS of a non-hibernating mammal (i.e., the rat), which underwent an experimental reversible deep hypothermia with suspended animation (i.e., ST; Cerri et al. 2013). Concerning the physiological function of the constitutive AT8-IR found in the ENS of normothermic rats, we can only hypothesize a link with some neuronal plasticity process (Wang and Mandelkow 2016). However, the overexpression of AT8-IR in the ENS during ST suggests the existence of a physiological mechanism, involved with an adaptive response of enteric neurons to extreme conditions (i.e., a very low core body temperature). The concomitant reduction of the number of nitrergic neurons in the ileum of ST animals can suggest a wide rearrangement of enteric neurons also involving the neurochemistry of these neurons, in addition to structural proteins. In conclusion, the strong parallelism of overall PP-Tau accumulation between ST (AT8), natural hibernation and AD in humans corroborates the possibility of a common mechanism underlying the cellular processes/modifications observed in all these cases.

## Electronic supplementary material

Below is the link to the electronic supplementary material.

Supplementary file1 (JPG 352 kb)
