# Supplementary figures and images for: Phosphorylated Tau protein in the myenteric plexus of the ileum and colon of normothermic rats and during synthetic torpor
Source: Cell Tissue Res. 2021 Jan 29;384(2):287–99. doi: 10.1007/s00441-020-03328-0 (PMC8141491; doi:10.1007/s00441-020-03328-0)

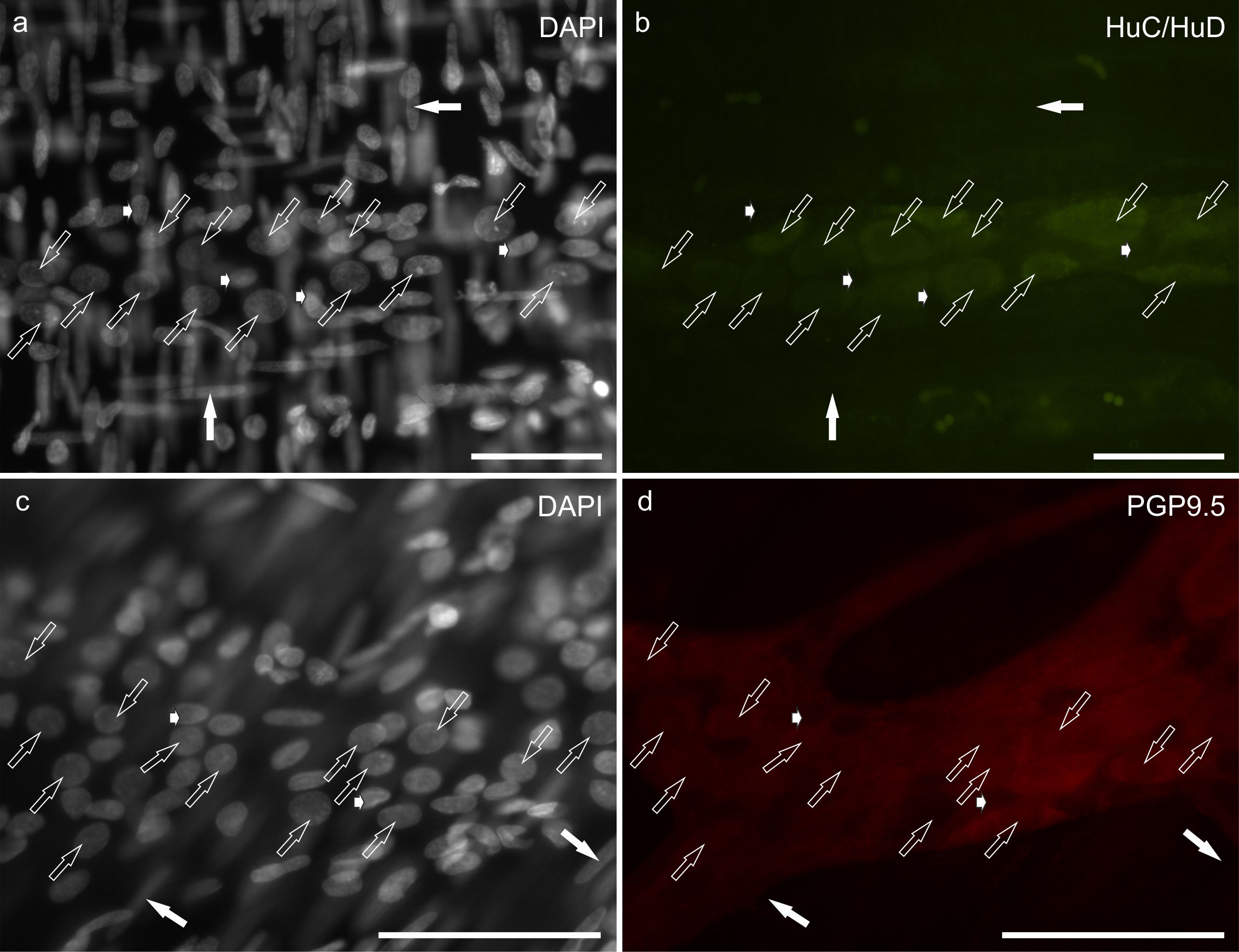

Supplement: Supplementary file 1 — Supplementary file1 (JPG 352 kb) [file 441_2020_3328_MOESM1_ESM.jpg]
